# Racial disparities with PRN medication usage in inpatient psychiatric treatment

**DOI:** 10.1038/s41537-024-00461-5

**Published:** 2024-04-13

**Authors:** Areef S. Kassam, Peter Karalis, Taline Aydinian, Anita Panjwani, Gabriel Martinez, Aaron Whiteman, Magdoline Daas, E. Ann Cunningham

**Affiliations:** 1 Community Health Network, Behavioral Health Department, Indianapolis, IN USA; 2 Harvard Medical School, Department of Psychiatry, Boston, MA USA; 3 Purdue University, Department of Nutrition Science, West Lafayette, IN USA; 4 Center on Aging and the Life Course, Purdue University, West Lafayette, IN USA

**Keywords:** Psychosis, Schizophrenia

## Abstract

Racial disparities in psychiatric diagnoses and treatment have significant public health implications, contributing to inequities in healthcare outcomes. We specifically examined racial disparities regarding pro re nata (PRN), or as needed, medications. Data from 14,616 encounters across 2019–2020 within Community Health Network’s inpatient psychiatric setting in Indianapolis, Indiana were included in this study. Due to the demographic sample size, analyses were narrowed to Black and White patients. Primary outcomes included comparisons across race for all PRN administrations and PRN administrations of antipsychotics vs. non-antipsychotics. Logistic regression was used to examine associations between race and PRN administrations by medication category, including all antipsychotics vs. non-antipsychotics overall, hydroxyzine, and lorazepam, independently. Significant differences in the percentage of administrations between Black and White patients were observed. Black patients received more PRN medications overall (71.0%) compared to White patients (67.7%) (*p* < 0.01). Further, while 17.7% of Black patients were administered PRN antipsychotics, this was true for only 8.2% of White patients (*p* < 0.001). When comparing antipsychotic PRNs with non-antipsychotic, hydroxyzine, and lorazepam PRNs, independently, Black patients were 58% (OR 1.58, *p* < 0.001), 109% (OR 2.09, *p* < 0.001), and 32% (OR 1.32, *p* < 0.001), more likely to receive antipsychotic PRNs, respectively, than White patients, controlling for sex, age, length of stay, and psychotic disorder diagnosis. Our study identifies yet another area of medical care with significant racial disparities. In this analysis of PRN medications during psychiatric admission, we identified significant differences in medication utilization by race. This information provides a basis for further investigation of disparities in patient-centered data.

## Introduction

It is well known both Psychiatry and Medicine have a dark history when it comes to marginalized populations and institutionalized racism^1–4^. In 2021, the American Psychiatric Association (APA) issued an apology to people of color for its role in perpetuating racism, discrimination, and racial hierarchy throughout its history. While an important step in addressing our role in history and the present day, this also begs the question—what can we now do as advocates and allies of communities that continue to be marginalized?

One example of such tangible disparities is the literature that reveals differences in the use of first-generation (FGAs) and second-generation antipsychotics (SGAs). SGAs, while more expensive, often give patients a better quality of life due to their improved side effect profile compared to their older counterparts and lower risk of extrapyramidal side effects. SGAs are thus generally viewed as the first-line choice for a variety of psychiatric conditions compared to their FGA counterparts. Interestingly, Black patients were more frequently prescribed FGAs and less frequently offered SGAs, while also being given higher doses of these agents compared to White peers^5^. Additionally, Black and minority patients are significantly more likely to be offered long-acting antipsychotic medications compared to white patients, suggestive of prescriber belief that certain ethnicities have decreased medication compliance^6^. This bias manifests in the exclusion of discussions on certain treatment plans due to provider assumptions on patients’ ability or likelihood to utilize the recommended regimen^7^.

Pro re nata (PRN), or as needed, antipsychotics are often used to calm individuals who are agitated and/or violent to the point of harm towards themselves or others. However, they are frequently used for common, more benign measures as well including sleep disturbance, anxiety, and impulsivity. We chose to focus on antipsychotic PRN medications as a marker of comparison when looking at racial discrepancies for several reasons: we were able to account and control for admission diagnosis, antipsychotic PRNs have the greatest risk for potential harm to a patient, and there is a clear clinical argument that antipsychotic PRN medication use is not a preferable treatment for non-psychotic patients.

It is widely shown in research that black patients have a three-fold increase in diagnosis of Schizophrenia. Psychiatrist Jonathan Metzl in his book *Protest Psychosis* discusses that unrest during the Civil Rights movement worsened the diagnostic disparity by strengthening societal stigmas at the time with black individuals being perceived as more agitated, paranoid, and aggressive^8^. This concern led to the foundation of this analysis is the role of ongoing societal stigmas that may continue to impact the treatment decision-making process. Racial discrepancies in the use of PRN medications in the inpatient setting have not yet been explored. As these outcomes have not been previously assessed in the inpatient setting, the aim of the current study is to examine whether racial disparities were present in the utilization of PRN medications within an inpatient, community-based psychiatric facility. We were most interested in antipsychotic PRN medications as well as the most used non-antipsychotic PRN medications within our facility, often ordered from our routine admission order set.

## Methods

### Patients

To collect admission and PRN medication administration data from Community Health Network’s 123-bed inpatient psychiatric facility in Indianapolis, Indiana, we utilized the Willow reporting system, an existing internal data collection system within the Network. We merged two separate reports—one on inpatient encounters and one on PRN administrations—to mitigate selection bias of the PRN administrations. Previous datasets at our institution had never incorporated race or ethnicity when examining inpatient encounters or PRN administrations and were modified to newly include demographic information. Both datasets spanned two calendar years, from 1/1/2019 to 12/31/2020.

We utilized racial categories that were self-disclosed by the patient and included the following categories: American Indian or Alaska Native, Asian, Biracial, Black or African American, Native Hawaiian or Other Pacific Islander, Other, Patient Declines, Two or More Races, and White. However, due to the low sample size of other races, we narrowed our analysis down only to White and Black patients. Ethnicity was categorized as Hispanic vs. Non-Hispanic White.

The inclusion criteria for the “encounters” dataset was all patient encounters at the psychiatric hospital during that time interval without any encounters excluded. The “PRN medications” dataset included every unique administration of PRN medications given across the two-year time span and included haloperidol, chlorpromazine, ziprasidone and olanzapine, and non-antipsychotics including, lorazepam, trazodone, diphenhydramine, and hydroxyzine. Thus, this dataset had potentially multiple data points for each patient. By cross-referencing the PRN administration dataset with the inpatient data, we were able to determine which specific PRN medication(s), if any, was/were given during the admission. Our final dataset of Black and White patients included a total of 14,616 admissions to our facility and 64,872 PRN administrations. The non-antipsychotics noted above are part of our facility’s routine admission order set which also includes PRN medications for headache, nausea, and extrapyramidal symptoms. These PRN orders are placed at the time of admission by the attending psychiatrist with a noted indication for use. They are able to be administered by nursing staff for those indications, as needed, based on patient presentation and nursing assessment.

### Prescribers

Community Health Network’s 123-bed facility is split up into 9 unique units which are separated and staffed to meet specific programmatic and diagnostic needs including affective disorders, psychotic disorders, child & adolescent, substance use disorders, and geriatrics. The facility is staffed by eight attending psychiatrists, three internal medicine physicians, rotating residents, as well as a wide variety of interdisciplinary staff including pharmacy, nursing, and social work. Patients are triaged through a crisis and access department before being assigned to a unit that best meets the needs of their presentation, however, there are often factors that limit this including but not limited to bed availability and unclear diagnostic impression. The nocturnist psychiatry team covers the facility during the evening and night hours.

### Diagnoses and medications

Psychotic disorders defined in this study included: Schizophrenia, Schizoaffective Disorder, Major Depressive Disorder with Psychotic Features, Bipolar Disorder with Psychotic Features, Unspecified Schizophrenia Spectrum Disorder, and Substance/Mood Induced Psychotic Disorders. The PRN administration records obtained included the following medications: antipsychotics (haloperidol, chlorpromazine, ziprasidone, and olanzapine) and non-antipsychotics including, hydroxyzine, lorazepam, trazodone, and diphenhydramine.

Hydroxyzine and lorazepam were of particular interest as they both have FDA indications for anxiety and were specifically examined in comparison to antipsychotics. Trazodone and diphenhydramine were not included in this study as the comparison focus was anxiety and agitation rather than the indications associated with trazodone and diphenhydramine.

### Statistical analysis

We reported descriptive characteristics of our study population, including the patient’s age, sex, race, ethnicity, and primary diagnosis. We reported differences among these characteristics by antipsychotic PRN administration, including *t*-tests for continuous variables and chi-square tests for dichotomous variables. We also conducted chi-squared analyses for associations between race and antipsychotic PRN use and stratified by psychotic disorder diagnoses. We further conducted hierarchical logistic regression modeling to control for confounding variables, including patient age, sex, length of stay, and psychotic disorder diagnosis. For these analyses, race was the independent variable, and the dependent variables included several patient-centered outcomes including exposure to antipsychotics vs. non-antipsychotics, antipsychotics vs. hydroxyzine, and antipsychotics vs. lorazepam. A Human Subjects Review submission was completed to the Community Health Network IRB, and an exemption was made that informed consent was not required.

## Results

We were able to determine a significant amount of demographic information pertaining to each inpatient admission including race, ethnicity, age, gender, and primary diagnosis. Of a total of 15,117 unique admissions to our Behavioral Health Pavilion, we included 14,616 in our study as these were patients who reported their race as Black or African American (21.4%) or White (78.6%) (Table 1). The mean age of the patients in our study was 35.8 years, 51% were male, 2.3% reported being of Hispanic origin, and 18.7% had a primary diagnosis of a psychotic disorder as defined by our criteria in the Methods section. As our main outcome was race comparisons for PRN administrations of antipsychotics vs. non-antipsychotics, we stratified the demographic characteristics by these categories. Compared to those who had exposure to PRNs but not antipsychotic PRNs, a greater percentage of those receiving antipsychotic PRNs were younger (32.2 years vs. 36.9 years, *p* < 0.001), male (56.9%, *p* < 0.001), Black (29.2%, *p* < 0.001), and diagnosed with a psychotic disorder (37.9%, *p* < 0.001). No statistical differences were found by ethnicity.

**Table 1:** Breakdown of demographics and primary diagnosis of each admission to our facility in years 2019 and 2020 by antipsychotic PRN administration (vs. other PRN administration).

		Antipsychotic PRN		
*n* (%)	Total (*N* = 14,616)	No (*N* = 11,055)	Yes (*N* = 3561)	*p*-value
Age, mean (SD)	35.8 (17.1)	36.9 (17.9)	32.2 (13.9)	<0.001
Sex				<0.001
Male	7457 (51.0)	5434 (49.1)	2023 (56.9)	
Female	7151 (49.0)	5621 (50.9)	1530 (43.1)	
Race				<0.001
White	11,494 (78.6)	8,973 (81.2)	2521 (70.8)	
Black	3122 (21.4)	2082 (18.8)	1040 (29.2)	
Ethnicity				0.29
Non-Hispanic	14,192 (97.7)	10,729 (97.6)	3463 (97.9)	
Hispanic	338 (2.3)	264 (2.4)	74 (2.1)	
Psychotic disorder				
No	11,896 (81.3)	9661 (87.4)	2208 (62.1)	<0.001
Yes	2739 (18.7)	1394 (12.6)	1345 (37.9)	

Note: Those receiving antipsychotic PRNs were not mutually exclusive of receiving other PRNs.

Examining inpatient encounters of all types of PRN medications by race, 67.8% of White patients vs. 71.0% of Black patients received at least one type of PRN medication compared to never having received a PRN (*p* < 0.01) (Table 2).

**Table 2:** Race comparison for admissions where a PRN medication was administered.

*n* (%)	Total (*N* = 14,616)	White (*N* = 11,494)	Black (*N* = 3122)	*p*-value
Never PRN	4611 (31.6)	3706 (32.2)	905 (29.0)	0.001
PRN	10,005 (68.5)	7788 (67.8)	2215 (71.0)	

Overall, a total of 64,872 PRNs were administered to patients at CHN across the two years and just over 10% of them were antipsychotics (Table 3). Stratifying PRN antipsychotic administrations by race, 8.2% of White patients were administered PRN antipsychotic medications while 17.7% of Black patients were administered these same medications (*p* < 0.001).

**Table 3:** Racial demographics compared across PRN medication administration class.

*n* (%)	Total (*N* = 64,872)	White (*N* = 50,778)	Black (*N* = 14,094)	*p*-value
Non-antipsychotic	58,241 (89.8)	46,634 (91.8)	11,607 (82.4)	<0.001
Antipsychotic	6631 (10.2)	11,607 (8.2)	2487 (17.7)	

The comparison in Table 3 does not take into account diagnosis, one of the biggest drivers in the selection of medication class for a PRN. Thus, this analysis (PRN administration of antipsychotics compared to non-antipsychotics) was further stratified by patients without a psychotic disorder (5.8% of White patients vs. 9.9% of Black patients, *p* < 0.001) and with a psychotic disorder (15.5% of White patients vs. 25.0% of Black patients, *p* < 0.001) (Table 4).

**Table 4:** Racial demographics compared across PRN medication administration class and stratified by primary admission diagnosis.

No psychotic disorder diagnosis				
*n* (%)	Total (*N* = 45,250) (100%)	White (*N* = 38,371) (84.8%)	Black (*N* = 6879) (15.2%)	*p*-value
Non-antipsychotic	42,347 (93.6)	36,152 (94.2)	6195 (90.1)	<0.001
Antipsychotic	2903 (6.4)	2219 (5.8)	684 (9.9)	
Psychotic disorder diagnosis				
*n* (%)	Total (*N* = 19,622) (100%)	White (*N* = 12,407) (63.2%)	Black (*N* = 7215) (36.8%)	*p*-value
Non-antipsychotic	15,894 (81.0)	10,482 (84.5)	5412 (75.0)	<0.001
Antipsychotic	3728 (19.0)	1925 (15.5)	1803 (25.0)	

Finally, we conducted several logistic regressions and for each, we show several layers of controlled variables: Model 1 reflects a crude odds ratio, and Model 2 controls for sex and age, length of stay, and diagnosis (psychotic disorder vs. non-psychotic disorders). The first analysis compares PRN administration by class, comparing antipsychotics to non-antipsychotics, and showed that Black patients were 58% more often treated with antipsychotic PRNs over non-antipsychotic PRNs compared to White patients (OR 1.58, *p* < 0.001) (Table 5). The second compares the usage of hydroxyzine to antipsychotics, showing Black patients were 109% more often treated with antipsychotic PRNs over hydroxyzine compared to White patients (OR 2.09, *p* < 0.001). Finally, the last analysis compares the usage of lorazepam to antipsychotics, and Black patients were 32% more often given antipsychotics over lorazepam compared to their White counterparts (OR 1.32, *p* < 0.001).

**Table 5:** PRN administrations by class (antipsychotics v. non-antipsychotics) between Black and White patients.

All PRN antipsychotics vs. all PRN non-antipsychotics		
Model 1 (*N* = 64,872)	Odds ratio	*p*-value
White	(reference)	-
Black	2.41 (2.29, 2.54)	<0.001
Model 2 (*N* = 64,872)		
White	(reference)	-
Black	1.58 (1.49, 1.67)	<0.001
All PRN antipsychotics vs. PRN hydroxyzine		
Model 1 (*N* = 30,102)	Odds ratio	*p*-value
White	(reference)	-
Black	3.17 (2.98, 3.37)	<0.001
Model 2 (*N* = 30,102)		
White	(reference)	-
Black	2.09 (1.95, 2.23)	<0.001
All PRN antipsychotics vs. PRN Lorazepam		
Model 1 (*N* = 15,713)	Odds ratio	*p*-value
White	(reference)	-
Black	2.00 (1.86, 2.14)	<0.001
Model 2 (*N* = 15,713)		
White	(reference)	-
Black	1.32 (1.21, 1.42)	<0.001

Model 1: univariate model with race.

Model 2: adjusted for patient sex, age, length of stay, and psychotic disorder diagnosis.

## Discussion

Our findings demonstrate statistically significant racial disparities regarding PRN medication usage in a community inpatient psychiatric unit. We were able to quantifiably show that our Black patients were more likely to receive a PRN medication than our White patients and that antipsychotic PRNs, specifically, were more likely to be chosen for Black patients even after controlling for sex, age, length of stay, and psychotic disorder diagnosis.

These findings have significant implications for addressing healthcare disparities and social determinants of health. Given the pattern of increased rates of diagnosis of schizophrenia and/or psychosis in the Black population compared to White peers, questions regarding both accuracy and implicit bias within clinical judgment have arisen^8^. In one prospective study of birth cohorts within the United States, it was found that Black patients had a three-fold increase in schizophrenia diagnosis compared to their White counterparts^8^. There have been several hypothesized reasons for this stark difference. Most commonly, it has been thought to be an interplay between epigenetics, trauma encountered through systematic oppression, and bias within the diagnostic process^4^. Implicit bias describes the phenomena where a person has a bias regarding groups of people that are non-deliberate, unconscious, and automatic within their thought patterns^9^. It has been shown that both medical students and psychiatrists collectively scored highly on Implicit Association Tests regarding racially charged behavior^9^. Participants had moderately strong associations of Black patients linked with concepts of psychosis and medication non-compliance, while there were strong associations of White patients with concepts of mood disorders^10^. This is consistent with evidence that Black individuals are more likely to be diagnosed with psychotic disorders while White individuals are more likely to be diagnosed with mood disorders^4^.

Our study findings were consistent with the literature in that Black patients were proportionally over-represented in the psychotic disorder cohort of patients, which in and of itself is an item that begs further exploration in future research. Our study attempts to control for psychotic disorders serving as a confounding variable in the logistic regression analysis, yet it still must be taken into consideration that the acuity of these different groups may intrinsically differ and thus is a potential limitation of the study. This study did not examine racial disparities in psychiatric diagnosis. However, it did examine differences in the use of PRN antipsychotics in patients with and without psychotic disorders. Statistically significant differences were noted in patients both with and without a psychotic disorder diagnosis. When considering the non-psychotic disorder cohort of the patients analyzed, we could posit that implicit bias may emerge in clinicians who may be quicker to assume that a Black patient is experiencing psychotic agitation. Antipsychotic PRNs in the inpatient setting for psychotic agitation are appropriately used to mitigate imminent harm and are avoided whenever possible due to the associated side effects and risks as previously enumerated.

Implicit bias translates into the quality of patient care and long-term health outcomes, speaking to an inequity of care based on race^9^. Our results show that Black patients have a statistically significant increased likelihood of being prescribed an antipsychotic PRN over hydroxyzine and lorazepam when compared to White patients. Agitation is a common indication for PRN medications and is the primary reason for antipsychotic PRNs to be given in an inpatient psychiatric setting. For psychotic agitation, antipsychotics have been shown to decrease aggression and psychotic symptoms^11^. Both hydroxyzine and lorazepam are options used to manage anxiety. In addition, lorazepam is often used as monotherapy or in conjunction with an antipsychotic for agitation management^12^ and was found to have equivalent efficacy to antipsychotics in a recent systematic review^13^. The suspicion that underlies the comparisons is that agitation is loosely defined in clinical settings where anxiety might likely be the underlying etiology for psychomotor activation yet is answered with antipsychotic or anti-manic agents. In such cases, particularly in non-psychotic psychiatric illness, anxiety medications such as lorazepam or hydroxyzine would be more appropriate options to prescribe and administer. Although some antipsychotics may aid anxiety, it is not their primary indication (apart from trifluoperazine which was not included in our study), and they have a far more unfavorable side effect profile. We posit antipsychotics are best utilized exclusively for behaviors relating to psychosis and/or agitation yet are being regularly prescribed as PRNs in a far more liberal fashion compared to more appropriate alternatives. This is a concern for all patients with a non-psychotic illness who are receiving unnecessary exposure to an antipsychotic. However, based on our findings, Black patients are alarmingly more likely to experience this inappropriate antipsychotic exposure.

In briefly underscoring the many concerns with inappropriate antipsychotic exposure, we must mention cardiovascular, musculoskeletal, and neurological adverse effects that can be caused even with just a few doses of antipsychotic medication. Cardiovascular complications from antipsychotic use range from tachycardia, blood pressure deviations, QTc prolongation, as well as, risk of death associated with cardiomyopathy and arrhythmias^14^. Risks of extrapyramidal side effects (EPS) such as akathisia, acute dystonia, Parkinsonism, and tardive dyskinesia (TD) can affect motor control and coordination and are highest in FGAs, most commonly haloperidol and fluphenazine^14^. TD is a feared form of EPS due to its relative irreversibility and involves involuntary movements of the mouth, tongue, lips, and face^14^. The development of neuroleptic malignant syndrome (NMS) is also a potentially life-threatening concern from antipsychotic administration and clinically manifests with autonomic instability, muscle rigidity, fever, and mental status changes^15^.

To explore reasons for the disparity, we hypothesize that clinicians may have an educational gap in properly identifying psychotic agitation and be more likely to misrepresent symptoms or behaviors when collaborating with the on-call physician. Literature shows that staff conceptualize antipsychotic treatment as serving to manage behavioral or emotional disturbances including anxiety, agitation, and compulsive behavior, and less to serve the treatment of underlying psychiatric disorders^16^. This educational gap in clinical staff may be further compounded by deficits in cultural humility and implicit bias, leading to further erroneous conclusions or tendencies when interacting with patients. Thus, if education were to be used as an intervention in correcting these disparities, it would need to marry cultural and structural bias along with enhancement of clinical skills in identifying root causes of psychiatric behaviors and agitation.

This study was strengthened by a large dataset spanning two years, collectively including 14,616 admissions and 64,872 PRN administrations. Furthermore, racial disparities within PRN medication usage have not yet been examined within the literature and are a novel research question worthy of further exploration. In our facility, the routine admission orders contain a variety of PRN medications with noted indications described earlier. It is at the discretion of the nursing team to administer these medications based on patient presentation and nursing assessment. Implicit bias can significantly impact patient-provider interactions, treatment decisions and adherence, and ultimately healthcare outcomes within the community. These findings should serve as a call to action for quality improvement within inpatient psychiatric settings. Department-wide implicit bias training can be an important structural change to help build education on how an individual can impact the care being delivered based on their own set of intrinsic beliefs and values. Education on PRN administration including a shared understanding of indications and presentations can ensure the reliability of assessment and proper utilization of PRNs. Additionally, new guidelines for PRN administration should be considered including process improvements such as prompts in electronic medical records to ensure proper administration use, limiting routine admission orders to include antipsychotics and require a real-time order from a provider, and development of less invasive interventions in de-escalating agitation to help trend health systems to more equitable care. It is important that healthcare institutions recognize that without structural support, inequitable care can continue to propagate.

Limitations of our study include solely examining PRNs and limiting the study to the inpatient psychiatric population. It would be beneficial to analyze these trends across a spectrum of settings, such as emergency rooms and medical units. This study took place at a single inpatient institution in the United States, and the findings that were presented here may not be generalizable to other facilities in the United States or other countries. This study was unable to include a thorough analysis of all racial identities for which data was collected. This narrower analysis of race was performed due to lacking statistical power in additional racial groups. However, an ability to include a more comprehensive patient population would benefit our understanding of how implicit bias might play into prescribing practices at large. While all PRN medications have associated set indications, it is a possible confounding element that these medications were given for a clinical presentation that was not in line with the indication marked on the electronic medical record^15,17–22^.

Additionally, although we were able to control for several important confounding variables, there are likely others that we are unable to account for such as baseline acuity of the patient, which could be incorporated into future research. The study and its findings are further limited in that we are unable to assess baseline acuity of initial assessment, severity of illness, and course of presentation during a patient’s inpatient stay. These factors may play a role in discrepancies between white and black patients resulting in PRN administration for agitation. Future studies could look at these factors as it cannot be discounted individual differences in symptom presentation at admission or during the course of hospitalization which could contribute to variations. There is known medical mistrust rooted in historical mistreatment in both research and medical treatment. While issues pertaining to racial disparities have gradually become more prevalent in the public eye, medical professionals are largely unaware of medication discrepancies, which generally are most notable when examined on a large scale. Further studies on PRN administration by unit, payor, zip code, method of administration, and dosage would also allow for a better understanding of socioeconomic implications of care that are more nuanced than race alone. An improved understanding of factors such as patient behaviors and illness characteristics that may impact prescriber behavior is needed and would better pave the way for interventions. Differential diagnosis of psychotic disorders by race and income level also requires additional research, both for understanding racial disparities in the use of antipsychotics and overall therapeutic outcomes.

## Conclusion

Our study identifies yet another area of medical care with significant racial disparities. In what we believe to be a novel examination of PRN medications during psychiatric admission, we identified significant differences in medication utilization by race. This information provides a basis for further investigation and uncovers disparities in patient-centered data. This study provides a foundation of great educational potential regarding education and addressing cultural, structural, and clinical bias which can lead to significant health disparities and outcomes.

## Data Availability

The authors confirm that the data supporting the findings of this study are available within the article. Raw data was generated at Community Health Network, and given its connection to protected patient health information it is not available for dissemination.
